# Deletion of PTPN22 improves effector and memory CD8^+^ T cell responses to tumors

**DOI:** 10.1172/jci.insight.127847

**Published:** 2019-08-22

**Authors:** Rebecca J. Brownlie, David Wright, Rose Zamoyska, Robert J. Salmond

**Affiliations:** 1 Leeds Institute of Medical Research at St. James’s, University of Leeds, Wellcome Trust Brenner Building, St. James’s University Hospital, Leeds, United Kingdom.; 2 Institute of Immunology and Infection Research, University of Edinburgh, Ashworth Laboratories, Edinburgh, United Kingdom.

**Keywords:** Immunology, Cancer immunotherapy, T cells

## Abstract

Adoptive T cell therapy (ACT) has been established as an efficacious methodology for the treatment of cancer. Identifying targets to enhance the antigen recognition, functional capacity, and longevity of T cells has the potential to broaden the applicability of these approaches in the clinic. We previously reported that targeting expression of phosphotyrosine phosphatase, nonreceptor type 22 (PTPN22) in effector CD8^+^ T cells enhances the efficacy of ACT for tumor clearance in mice. In the current work, we demonstrate that, upon ACT, PTPN22-deficient effector CD8^+^ T cells afforded greater protection against tumors expressing very low-affinity antigen but did not survive long term in vivo. Persistence of CD8^+^ T cells following tumor clearance was improved by ACT of memory phenotype cells that have a distinct metabolic phenotype, as compared with effector T cells. Importantly, PTPN22-deficient T cells have comparable capacity to form long-lived memory cells in vivo but enhanced antitumor activity in vivo and effector responses ex vivo. These findings provide key insights into the regulation of effector and memory T cell responses in vivo and indicate that PTPN22 is a rational target to improve ACT for cancer.

## Abstract 2

Deletion of the inhibitory phosphatase PTPN22 markedly improves both effector and memory CD8 T cells responses to tumors bearing low affinity antigens.

## Introduction

T cell–directed immunotherapy has delivered remarkable therapeutic benefits in cancer patients. For example, the use of function-blocking antibodies to immune checkpoints programmed death 1 (PD-1) and cytotoxic T lymphocyte antigen 4 (CTLA-4) has proven efficacious in otherwise untreatable and aggressive cancers, such as malignant melanoma (1–3). Nonetheless, checkpoint inhibition induces complete and durable therapeutic responses in only a small minority of patients and is completely dependent on the existence and functionality of preexisting tumor-reactive host T cells. Adoptive cell transfer of genetically engineered tumor-reactive T cells has long been touted as an alternative approach to cure and provide long-lasting protection from the reoccurrence of cancer (4, 5). Indeed, CD19-directed chimeric antigen receptor (CAR) T cell therapy is effective in curing otherwise intractable acute lymphoblastic lymphoma in up to 90% of pediatric patients (6). However, the toxicity associated with CAR T cell therapies means that there are substantial hurdles to overcome to improve the safety and wider applicability of these approaches in the clinic (7).

Potential alternatives to CAR T cell therapy include infusion of patients with autologous ex vivo–expanded tumor-infiltrating lymphocytes or T cells engineered to express tumor antigen-specific conventional T cell receptors (TCR). Furthermore, genetic manipulation of T cells to influence effector function, longevity, and metabolic capacity can be combined with expression of tumor-reactive TCRs to improve the outcome of such approaches. Previously, we determined that CD8^+^ T cells lacking the autoimmunity-associated inhibitory tyrosine phosphatase PTPN22 had enhanced capacity to clear tumors in mice (8, 9). The enhanced antitumor responses of PTPN22-deficient T cells were associated with elevated reactivity to low-affinity tumor antigens and a decreased susceptibility to inhibition by tumor-derived TGF-β (8).

A key consideration for ACT approaches to cancer therapy is to determine which T cell phenotype provides optimal long-lived protection. Consensus is emerging that terminally differentiated effector T cells provide only short-lived protection in cancer models (10–12). The role of PTPN22 in regulating memory T cell responses to tumors is unknown. In the current work, we show that, while *Ptpn22^–/–^* effector CTLs provide enhanced protection against tumors expressing very low-affinity antigens, neither the control nor the *Ptpn22^–/–^* effector T cells persist in vivo. By contrast, control and *Ptpn22^–/–^* memory phenotype CD8^+^ T cells were similarly long lived upon ACT. Increased longevity of control and *Ptpn22^–/–^* memory phenotype cells was associated with altered cellular metabolism, including enhanced mitochondrial spare respiratory capacity (SRC) and decreased glycolytic flux, compared with effector T cells. Importantly, upon transfer to naive recipient mice, very low numbers of long-lived *Ptpn22*^–/–^ memory T cells provided enhanced protection from tumor challenge, as compared with control cells. Strikingly, *Ptpn22^–/–^,* but not control, memory phenotype T cell ACT could completely protect mice from low-affinity antigen-bearing tumors when transferred to hosts 2–4 weeks prior to tumor implantation. Together, these experiments have determined that deletion of PTPN22 represents a rational approach to enhance the functional capacity of both short-lived effector and long-lived memory T cells in antitumor immunity.

## Results

### Ptpn22^–/–^ CTLs mediate enhanced clearance of low-affinity tumors.

**Figure 1:**
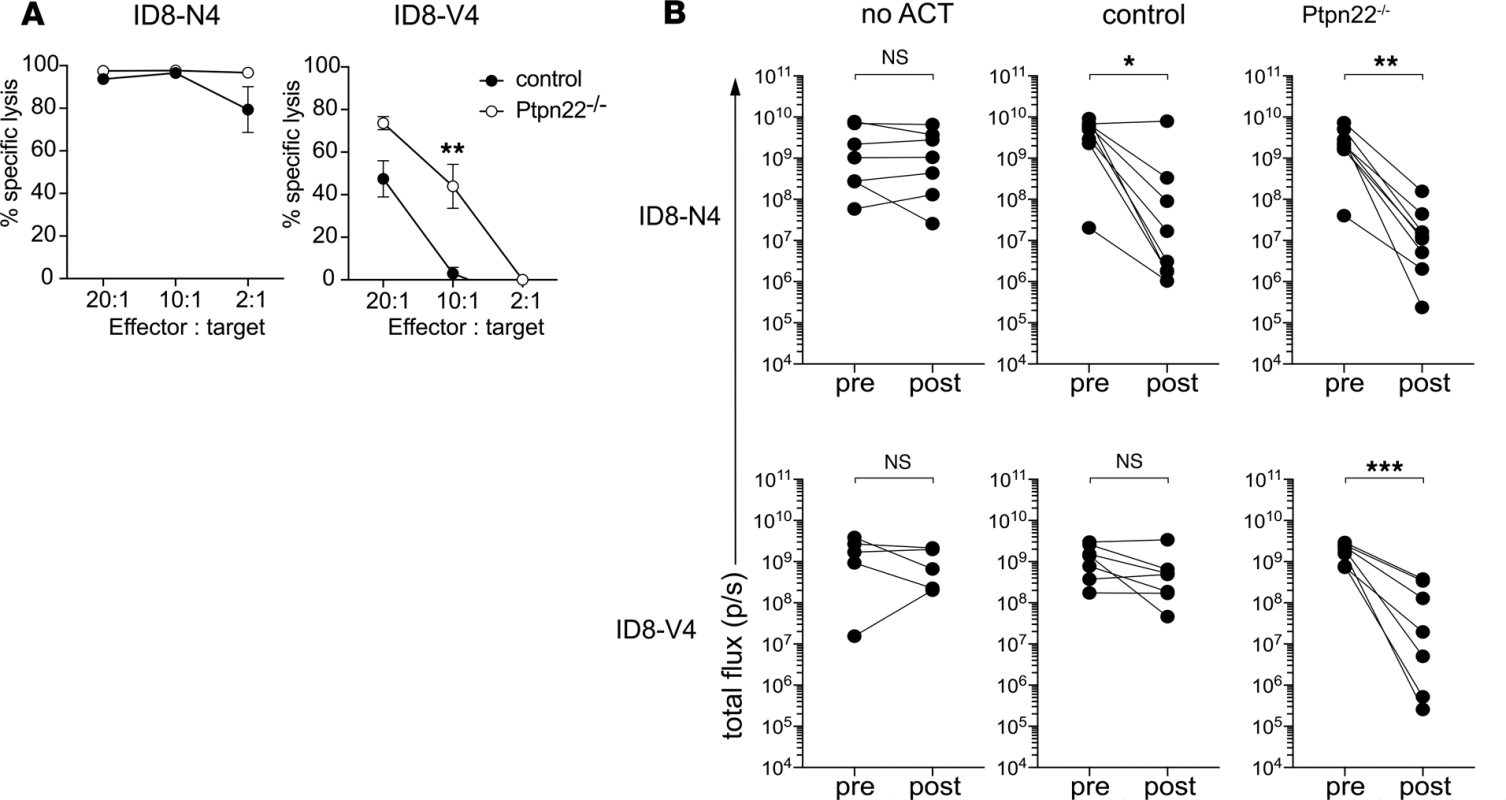
Effector CTLs deficient in PTPN22 kill tumor cells expressing low-affinity antigen more efficiently. (**A**) Effector control and *Ptpn22^–/–^* OT1 CTLs were assessed for their capacity to kill target ID8-fLuc cells expressing high- (N4) or extremely low-affinity (V4) antigen in an in vitro killing assay. ID8 cell death was measured by a decrease in luminescence, as assessed by IVIS. Graphs show the percentage specific lysis at various effector-to-target ratios. Control and *Ptpn22^–/–^* CTLs were both able to effectively kill ID8-N4-fLuc cells, whereas *Ptpn22^–/–^* CTLs were more effective than control CTLs at killing ID8-V4-fLuc targets. **P < 0.01, as determined by 2-way ANOVA. Effector, CTLs; targets, ID8 cells. (**B**) Groups (*n* = 7) of C57BL/6J mice were injected i.p. with 5 × 10^6^ ID8-N4-fLuc or ID8-V4-fLuc and assessed for tumor establishment on day 5 (pretreatment) by bioluminescence imaging. On day 6, groups of mice received no cells or 1 × 10^7^ effector control or *Ptpn22^–/–^* OT1 CTLs i.p. Graphs show the bioluminescence signal intensity of all mice on day 5 (1 day prior to ACT) and day 18 (12 days after ACT). Both control and *Ptpn22^–/–^* CTLs were able to suppress growth of ID8-N4 tumors, while only *Ptpn22^–/–^* CTLs could significantly suppress the establishment of ID8-V4 tumors. *P < 0.05, **P < 0.01, ***P < 0.001, as determined by Student’s paired *t* test.

CD8^+^ T cells mediate anticancer responses directly, by targeting and killing malignant cells, or indirectly, through the production of inflammatory cytokines (13). Our previous experiments determined an enhanced capacity of *Ptpn22*^–/–^ CD8^+^ T cells to produce IFN-γ and TNF in response to low-affinity peptide antigens (14, 15) and mediate tumor rejection upon ACT (8). We sought to determine the effect of PTPN22 deficiency on the capacity of CTLs to directly mediate target cell killing in response to strong and weak tumor-associated antigens (TAAs). The OVA-specific H2-K^b^–restricted OT-1 TCR transgenic system has been used extensively to investigate the effect of antigen affinity on T cell selection in the thymus and effector T cell responses (16–18). Control and *Ptpn22^–/–^* OT-1 T cells were activated with cognate SIINFEKL (N4) peptide for 2 days and then expanded and differentiated in a high dose of IL-2 for 4 days to generate inflammatory effector CTLs. ID8 ovarian carcinoma cells (19) expressing high-affinity N4 (*K_D_* for OT-1 TCR = 54 μM; ref. 17) or very low-affinity SIIVFEKL (V4; *K_D_* > 1 mM) OVA variants were used as targets cells. Control and *Ptpn22^–/–^* CTLs were equally effective in killing high-affinity ID8-N4 tumor cells (Figure 1A). By contrast, low-affinity ID8-V4 targets were killed much more efficiently by *Ptpn22^–/–^* CTLs as compared with control CTLs (Figure 1A). Consistent with the results of in vitro killing assays, control CTL ACT was sufficient to mediate a significant reduction in tumor burden in recipient mice bearing established high-affinity ID8-N4 but not low-affinity ID8-V4 intraperitoneal tumors (Figure 1B). Importantly, *Ptpn22^–/–^* effector CTL ACT enabled tumor clearance in response to both strong N4 and very weak V4 TAAs (Figure 1B). Previous studies have shown that TCR triggering influences expression of inhibitory phosphatases (15); thus, it was of interest to determine the levels of PTPN22 following stimulation of OT-1 T cells with weak and strong agonist peptides. Western blot analysis showed that levels of PTPN22 expression were elevated following stimulation of cells with high-affinity N4 as compared with low-affinity SIITFEKL (Supplemental Figure 1; supplemental material available online with this article; https://doi.org/10.1172/jci.insight.127847DS1).

### Memory phenotype, but not effector CTLs, persist after tumor clearance in vivo.

**Figure 2:**
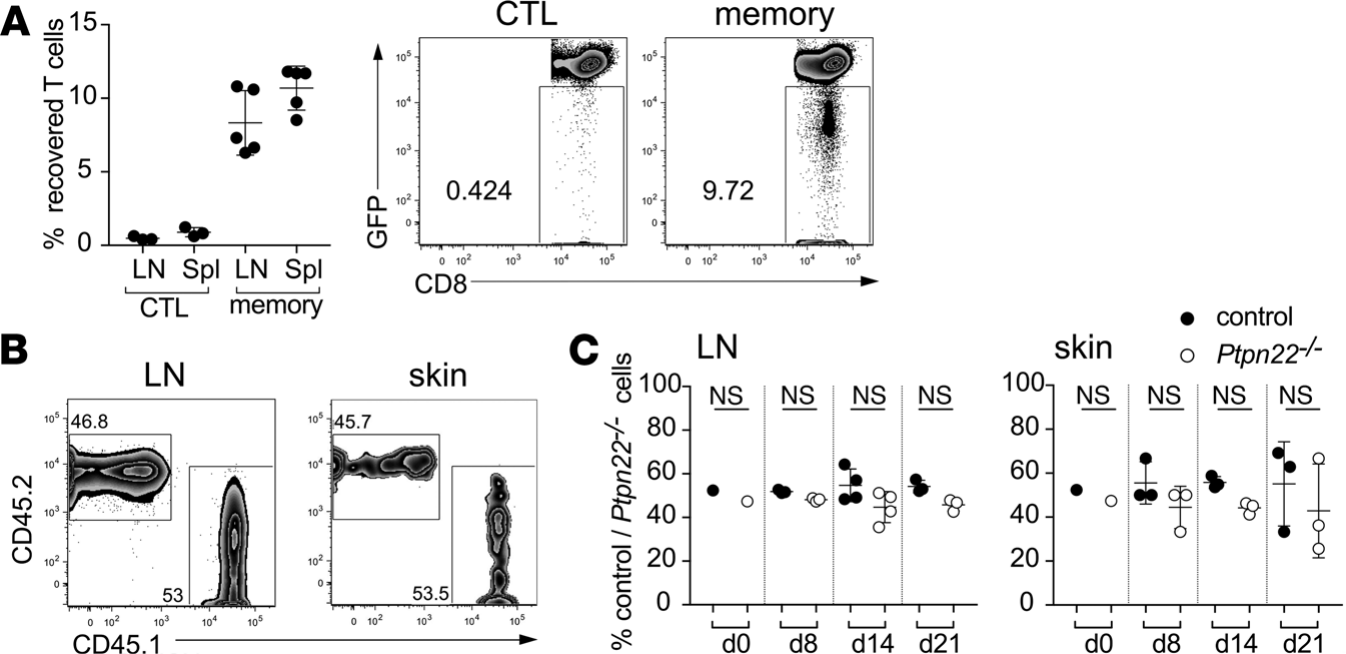
Ptpn22 does not effect the increased survival of memory versus effector CTLs in vivo*.* (**A**) On day 0, groups (*n* = 5) of C57BL/6 UbC-GFP hosts were injected with 1 × 10^6^ EL4-N4 cells s.c. On day 6 after EL4 injection, groups of mice were injected with a mix of control and *Ptpn22^–/–^* effector CTLs or memory cells i.v. The recovery of these cells within LN and spleen populations was assessed 3 weeks after injection (day 27) by FACS, and they were identified as GFP-ve, CD8β^+^ T cells. Example dot plots are shown and dots on the graph represent individual animals. The proportion of donor CTLs recovered within the total CD8^+^ population was extremely low (<0.5%); in contrast, the proportion of memory cells was approximately 10% of total CD8^+^ T cells. (**B** and **C**) C57BL/6 UbC-GFP hosts (*n* = 10) were injected with 1 × 10^6^ EL4-OVA cells s.c. (day = –6). After 6 days all mice were injected with an equal mix of control and *Ptpn22^–/–^* memory cells (5 × 10^6^ of each) (day = 0), and groups of mice (*n* = 3/4) were assessed at day 8, day 14, and day 21 after memory cell injection by FACS. Example dot pots (from d = 21) are shown (**B**), and graphs (**C**) show the proportion of recovered control versus *Ptpn22^–/–^* memory cells within the LN and skin at the various time points. The ratio of control/Ptpn22^–/–^ memory cells remains approximately 1:1 throughout the time course.

To determine the longevity of effector CTLs following tumor clearance in vivo, we cotransferred control and PTPN22-deficient cells to transgenic C57BL/6-Ubc-GFP recipient mice bearing high-affinity EL4-OVA s.c. lymphomas. Ubc-GFP mice have ubiquitous transgenic expression of GFP (20), enabling GFP^–^ donor T cells to be easily distinguished from host T cells. When assessed 3 weeks after transfer, GFP^–^ donor CTLs could not be reliably detected in lymph nodes of Ubc-GFP recipient mice (Figure 2A). Previous studies have defined a critical role for CD28 costimulation during T cell priming for the formation of long-lived memory T cell populations (21). Furthermore, in vitro culture in the presence of IL-15, rather than IL-2, favors the differentiation of memory phenotype CD8^+^ T cells (22). Therefore, we incorporated CD28 costimulation and IL-15 culture into our in vitro differentiation model to generate memory phenotype OT-1 T cells for ACT to EL4-OVA tumor-bearing mice. Importantly, and in contrast to effector CTLs, control memory phenotype OT-1 T cells were readily detectable 3 weeks after transfer in lymph nodes of Ubc-GFP host mice (Figure 2A). Cotransfer experiments were performed to compare the ability of control Ly5.1^+^ and *Ptpn22^–/–^* Ly5.2^+^ memory phenotype CD8^+^ T cells to persist in tumor-bearing hosts. Cotransfer of memory phenotype control and *Ptpn22^–/–^* CD8^+^ cells cleared s.c. EL4-OVA tumors, while the ratio of Ly5.1^+^/Ly5.2^+^ donor cells in lymph nodes and at the tumor site (skin) was assessed weekly by ex vivo FACS analysis (Figure 2, B and C). The results showed that the ratio of control/*Ptpn22^–/–^* T cells remained approximately 1:1 for the duration of the experiment, indicating that PTPN22 deficiency does not impinge on T cell survival and longevity following tumor clearance.

### PTPN22 limits cytokine production and target cell killing of memory phenotype T cells.

**Figure 3:**
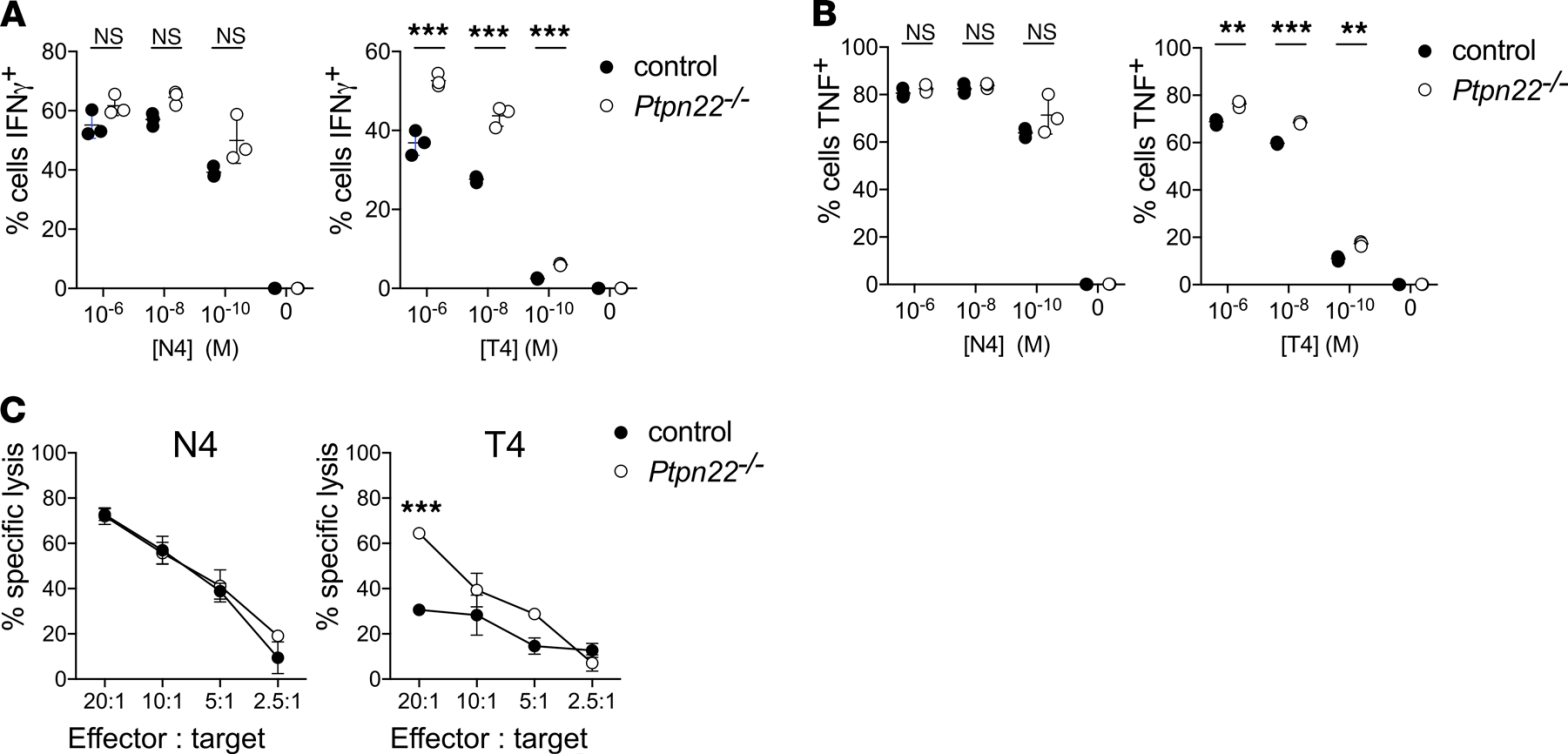
Ptpn22 deficiency improves cytokine production and killing capacity in memory cells stimulated with weak affinity antigens. Control and *Ptpn22^–/–^* memory phenotype cells were generated in vitro for 6 days before phenotypic and functional analysis. Control and *Ptpn22^–/–^* memory cells were restimulated with varying concentrations of high-affinity (N4) and low-affinity (T4) peptide for 4 hours before intracellular staining for IFN and TNF. Dots on the graphs show the frequency of IFN-γ^+^ (**A**) and TNF^+^ (**B**) cells within the population of memory cells generated from 3 individual mice. (**C**) Control and *Ptpn22^–/–^* memory phenotype cells were assessed for their capacity to kill target ID8-fLuc cells expressing high- (N4) or low-affinity (T4) antigen in an in vitro killing assay. ID8-fLuc cell death was measured by a decrease in luminescence, as assessed by IVIS. Graphs show the percentage specific lysis at various effector-to-target ratios. Ptpn22 deficiency increases the killing capacity and frequency of IFN-γ^+^ and TNF^+^ cells upon restimulation in response to weak affinity antigens. Effector, CTLs; targets, ID8 cells. **P < 0.01, ***P < 0.001, as determined by 2-way ANOVA.

Our data indicated that PTPN22 did not adversely influence T cell memory formation following ACT. Further experiments were undertaken to define the consequence of PTPN22 deficiency on the functional capacities of memory phenotype T cells. Intracellular staining and FACS analysis demonstrated that, as compared with memory phenotype cells, effector phenotype T cells typically expressed lower levels of CD62L and higher levels of cell surface receptors CD25, CD44, CD69, and CD71; transcription factors Tbet, Eomesodermin, and IRF4; and cytolytic effector protein granzyme B (Supplemental Figure 2). However, *Ptpn22* deficiency did not effect expression of these proteins in either effector or memory CD8^+^ T cell populations (Supplemental Figure 2). However, restimulation of memory phenotype OT-1 T cells and analysis of IFN-γ and TNF production revealed a striking difference in the capacity of PTPN22-deficient cells to respond to low-affinity OVA-T4 peptide (*K_D_* = 444 μM; ref. 17) but not OVA-N4 (Figure 3, A and B). Furthermore, while control and *Ptpn22^–/–^* memory phenotype T cells were effective in killing high-affinity ID8-N4 targets in vitro, *Ptpn22^–/–^* cells had a markedly improved capacity to kill low-affinity ID8-T4 cells (Figure 3C). Therefore, and similar to the case for effector CTLs, PTPN22 limits the functional capacity of memory phenotype CD8^+^ T cells in vitro.

**Figure 4:**
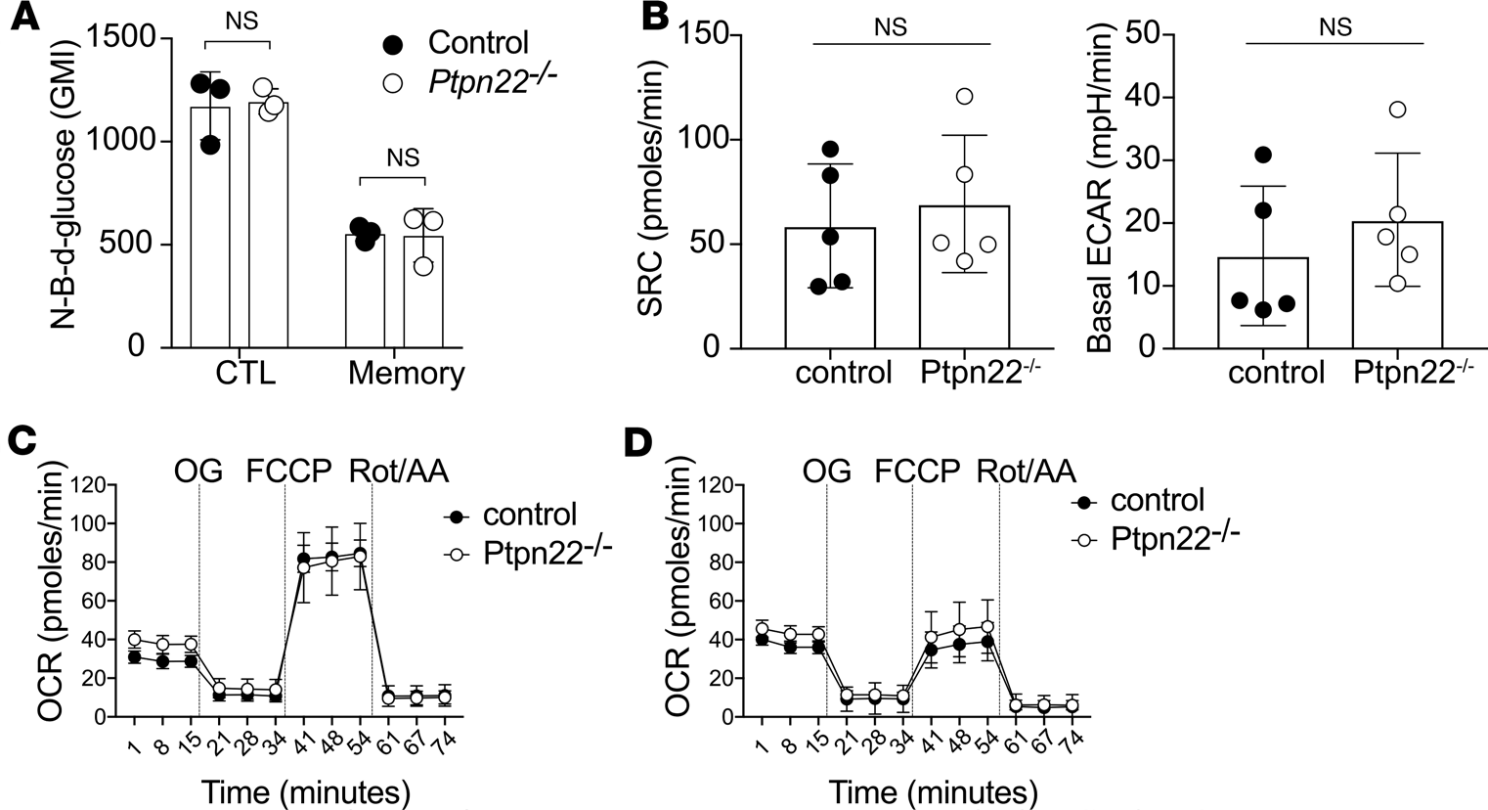
Effector and memory phenotype CTLs have distinct metabolic profiles, but PTPN22 does not effect these. Effector and memory phenotype OT-1 cells were differentiated for 6 days. On day6, glucose uptake was assessed using fluorescent NB-*d*-glucose (NBDG) and FACS. Effector CTL had higher uptake than memory phenotype cells (**A**), but there was no difference between control and *Ptpn22^–/–^* cells. Mitostress test Seahorse analyses were performed (**B**–**D**). Analysis of spare respiratory capacity (maximal stressed OCR – basal OCR) and basal ECAR (i.e., glycolysis) showed similar profiles for control and *Ptpn22^–/–^* memory phenotype cells (**B**). Full Seahorse profiles for memory (**C**) and effector (**D**) CTLs show no effect of PTPN22 deficiency on OXPHOS. NS, not significant; as determined using Student’s *t* test.

A wealth of data have indicated that the regulation of cellular metabolic pathways is key to establishing T cell phenotype. Previous studies determined that memory T cells have enhanced mitochondrial SRC and reduced glycolytic flux as compared with effector T cells (21, 23). Our data indicated that both effector and memory phenotype T cell functional capacity (cytokine production and killing capacity) were negatively regulated by PTPN22; therefore, we wished to test whether these phenotypes were associated with altered cellular metabolism. Uptake of the fluorescent glucose analog 2-(N-(7-nitrobenz-2-oxa-1,3-diazol-4-yl)amino)-2-deoxyglucose (2-NBDG) by in vitro–generated effector OT-1 CTLs was substantially higher than that of memory phenotype OT-1 cells (Figure 4A). However, PTPN22 deficiency did not effect 2-NBDG uptake in either cell population. Analysis of oxygen consumption rate (OCR) using a Seahorse XFe96 Analyzer showed that *PTPN22* deficiency did not affect SRC (Figure 4B) or mitochondrial metabolism in either memory phenotype (Figure 4C) or effector (Figure 4D) OT-1 T cells. Furthermore, basal glycolytic flux, as assessed by extracellular acidification rate (ECAR), was similar for control and *Ptpn22^–/–^* memory phenotype OT-1 T cells (Figure 4B). Therefore, PTPN22 deficiency does not impinge upon the 2 major ATP-producing pathways in effector or memory phenotype T cells.

### PTPN22 deficiency improves memory T cell–mediated tumor clearance.

**Figure 5:**
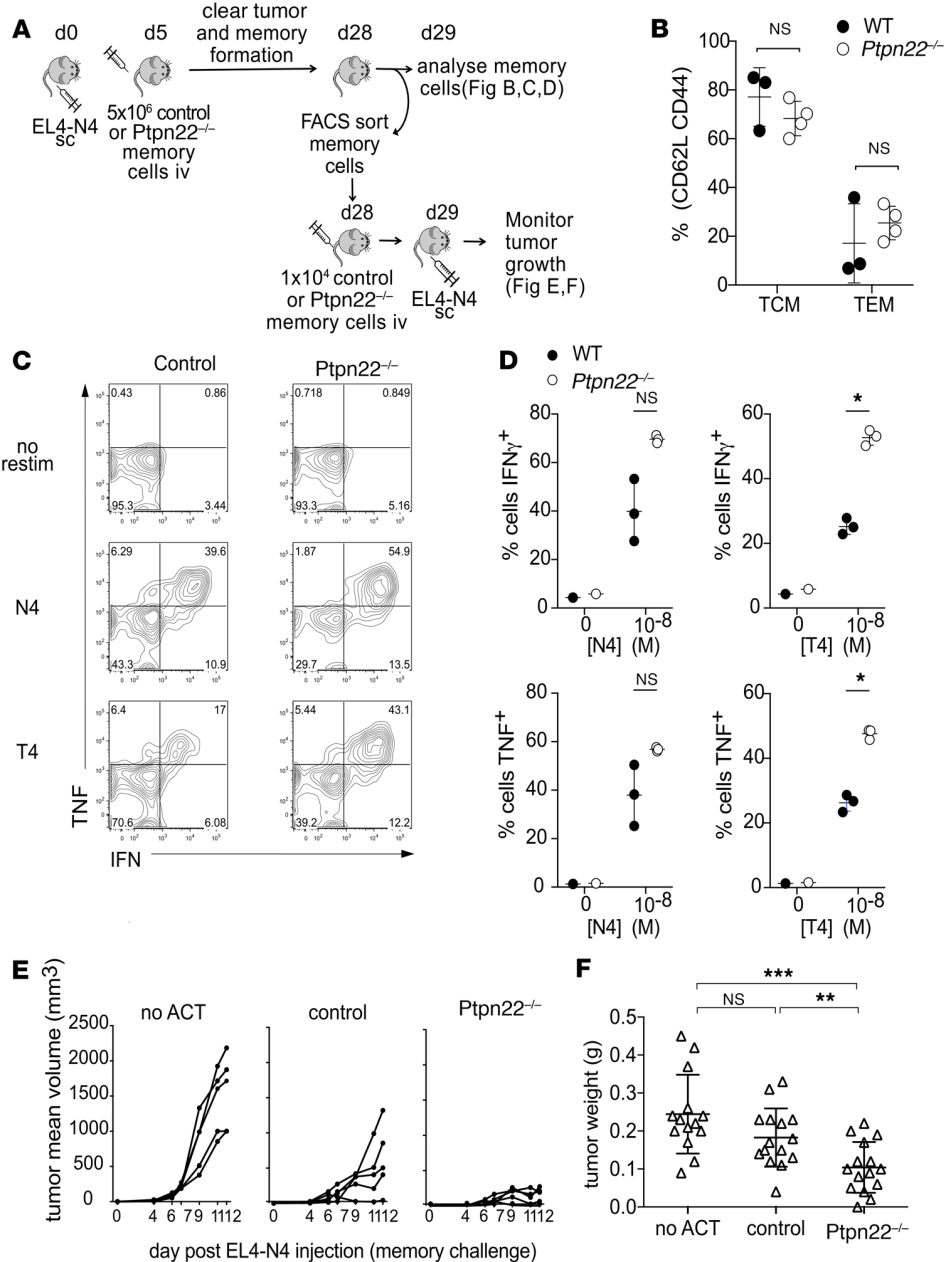
PTPN22 deficiency improves T cell memory to tumors. (**A**) Scheme of experimental design. (**B**) Groups of C57BL/6-UbC-GFP hosts were injected with 1 × 10^6^ EL4-N4 s.c. (day 0) before transfer of 5 × 10^6^ control or *Ptpn22^–/–^* memory cells i.v. on day 5. On day 29, LNs were harvested from animals and the proportion of central memory (TCM: CD44^+^CD62L^+^) and effector memory (TEM: CD44^+^CD62L^–^) cells within the population of recovered memory cells (CD8β^+^GFP^–^) was quantified by FACS. (**C** and **D**) LN cells were also restimulated with varying concentrations of high-affinity (N4) and low-affinity (T4) peptide for 4 hours before intracellular staining for IFN-γ and TNF. Dots on the graphs show the frequency of IFN-γ^+^ and TNF^+^ cells within the population of recovered memory cells (day 29) (CD8β^+^GFP^–^). (**E** and **F**) Groups (*n* = 5) of C57BL6-UbC-GFP hosts were injected with 1 × 10^6^ EL4-N4 s.c. before being injected with 5 × 10^6^ control or *Ptpn22^–/–^* memory phenotype cells i.v. on day 5. On day 28, LNs and spleens were collected, and memory OT-1 cells were sorted by FACS (CD8β^+^, CD44^+^, GFP^–^). 1 × 10^4^ sorted control or *Ptpn22^–/–^* cells were transferred i.v. into additional groups of C57BL/6-UbC-GFP hosts (day 28), which were injected with 1 × 10^6^ EL4-N4 the subsequent day (day 29). Control mice received no memory cell ACT. Tumor growth was monitored by caliper measurement, as shown for individual mice from 1 experiment (**E**), and tumor weight was measured at the end of the experiment (day 12 after EL4-N4 injection). Each dot represents an individual mouse and represents data from 3 pooled experiments **(F**). *P < 0.05, **P < 0.01, ***P < 0.001, as determined by Student’s *t* test (**B** and **D**) or 1-way ANOVA with Tukey’s multiple comparisons test (**F**).

In order to test whether PTPN22 deficiency in memory T cells afforded protection against tumors in vivo, we used a serial ACT approach (Figure 5A). Ubc-GFP mice were injected with s.c. EL4-OVA lymphoma cells and then infused i.v. with large numbers (i.e., 10^7^) of control or *Ptpn22^–/–^* memory phenotype OT-1 T cells, which were sufficient to clear the primary tumor (Supplemental Figure 3). Following 21–28 days, long-lived memory OT-1 T cells were phenotyped ex vivo. The majority of both control (~80%) and *Ptpn22^–/–^* (~70%) OT-1 T cells had a CD44^hi^CD62L^hi^ central memory–like (Tcm-like) phenotype (Figure 5B). There was a nonsignificant trend toward an elevated proportion of *Ptpn22^–/–^* cells having a CD44^hi^CD62L^low^ effector memory phenotype (Figure 5B). Restimulation with low-affinity OVA-T4 variant peptides revealed enhanced IFN-γ and TNF production by PTPN22-deficient cells (Figure 5, C and D), consistent with the phenotype of cells prior to ACT (Figure 3C). Furthermore, memory OT-1 T cells were FACS sorted to high purity and adoptively transferred i.v. to C57BL/6J hosts. Groups of mice were challenged with s.c. EL4-OVA cells and tumor mass was assessed as wet weight at experimental endpoints. Strikingly, low numbers (i.e., 10^4^) of adoptively transferred *Ptpn22^–/–^*, but not control, memory T cells significantly reduced tumor burden in recipient mice (Figure 5, E and F).

**Figure 6:**
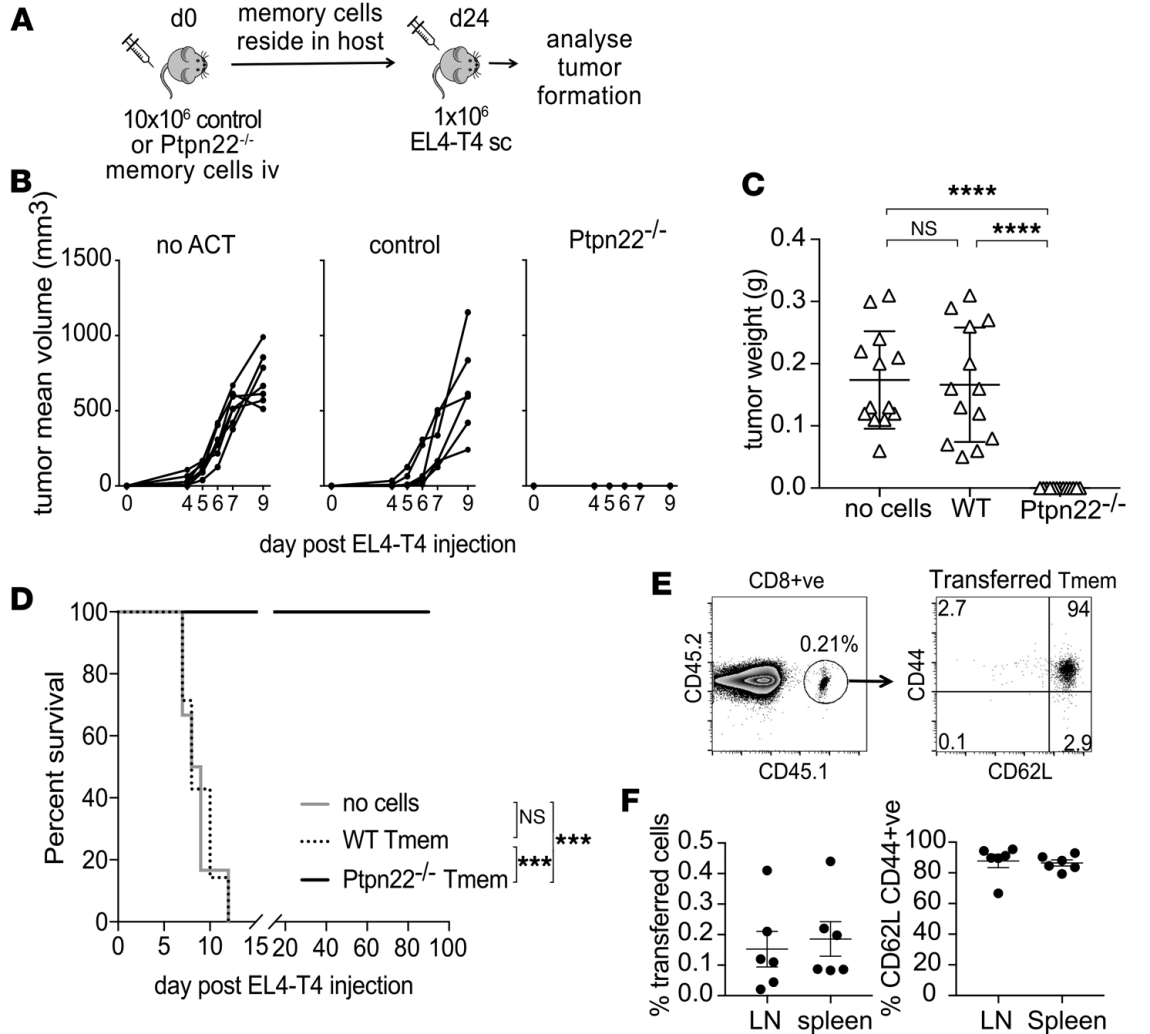
PTPN22-deficient memory T cells prevent growth of tumors expressing weak antigen. (**A**) Scheme of experimental design. (**B**) Groups of C57BL/6J hosts were injected with 10 × 10^6^ in vitro–generated control or *Ptpn22^–/–^* memory cells i.v. on day 0. On day 24, 1 × 10^6^ EL4 cells loaded with T4 peptide were injected i.v. Tumor growth was monitored by caliper measurement, as shown for individual mice from 1 experiment (**B**), and tumor weight was measured at the end of the experiment (day 9 after EL4-T4 injection, when tumors in control mice reached a maximum-allowed diameter of 15 mm) (**C**). Each dot represents an individual mouse from 2 repeated experiments (**C**). ****P < 0.0001, as determined by 1-way ANOVA with Tukey’s multiple comparison test. (**D**) Groups of C57BL/6J hosts (*n* = 7/group) were injected with 10 × 10^6^ in vitro–generated control or *Ptpn22^–/–^* memory cells i.v. on day 0. Fourteen days after T cell ACT, 1 × 10^6^ EL4 cells loaded with T4 peptide were injected s.c. Tumor growth was monitored by caliper measurement at least 2 times a week for up 90 days after EL4-T4 injection. When tumor diameter exceeded 10 mm, mice were removed from the study. ***P < 0.0001, as determined by χ^2^ and log-rank test. (**E** and **F**) LNs and spleens were removed from all mice that had received *Ptpn22^–/–^* T memory cells at the end of the study (day 90 after EL4-T4 injection) and assessed for the presence of transferred T cells by FACS. Representative dot plots shows the percentage of *Ptpn22^–/–^* T memory cells (CD8^+^CD45.1^+^CD45.2^+^) and expression of CD62L and CD44. (**F**) Dots on the corresponding graphs represent values from individual animals.

We tested the ability of control and *Ptpn22^–/–^* memory T cell to mediate protection against low-affinity tumors in vivo. Control or *Ptpn22^–/–^* memory phenotype T cells were transferred to host mice, and, following engraftment for 24 days, hosts were challenged with OVA-T4–bearing EL4 tumors (Figure 6A). Hosts receiving either no ACT or control T cell ACT developed tumors at a similar rate (Figure 6B) and of a similar size (Figure 6C). Remarkably, ACT of *Ptpn22^–/–^* memory phenotype T cells, 24 days prior to tumor challenge, completely prevented the development of EL4-OVA-T4 tumors in 100% of mice tested (Figure 6, B and C, 13 of 13 mice in 2 experiments). Finally, we tested the longevity of protection afforded by ACT of *Ptpn22^–/–^* memory phenotype cells. Whereas hosts receiving either no ACT or control ACT succumbed to OVA-T4–bearing tumors within 12 days after challenge, mice (*n* = 7) receiving *Ptpn22^–/–^* memory phenotype cells remained entirely tumor free for at least 90 days following tumor cell injection (Figure 6D). Flow cytometry analysis of host lymph nodes and spleens determined that a population of transferred central memory phenotype *Ptpn22^–/–^* CD8^+^CD44^hi^CD62L^+^ T cells could be detected at the experimental endpoint (day 90 after tumor challenge and day 104 after ACT) (Figure 6, E and F). Together, these data indicate that PTPN22-deficient CD8^+^ memory T cells retain their enhanced functional characteristics and are superior in their capacity to provide long-lived protection from tumor growth in response to both high- and low-affinity tumor antigens in vivo, as compared with control T cells.

## Discussion

Effective and long-lived immune protection from malignancies requires both an immediate antitumor effector response and maintenance of a population of protective memory T cells. In the current study, we have demonstrated that PTPN22 is a key negative regulator of both effector and memory T cell responses to tumors. Effector and memory phenotype *Ptpn22^–/–^* CD8^+^ T cells have enhanced cytolytic capacity and elevated cytokine production in response to low-affinity TAAs, as compared with control counterparts. Furthermore, long-lived *Ptpn22^–/–^* memory T cells retain their enhanced capacity to control tumor growth in vivo.

A majority of tumor-associated antigens that have been targeted in the clinic for immunotherapy are aberrantly expressed self-antigens (24). These antigens are expressed during development and, as a result of selection processes in the thymus and periphery, the responsive endogenous T cell repertoire is limited to cells expressing low-affinity TCRs (25). Expression of high-affinity CARs enables autologous T cells to respond to surface-exposed TAAs but not against intracellular antigens that are processed and presented via MHC molecules. Therefore, expression of conventional TCRs may be preferable for T cell ACT in some circumstances. Furthermore, genetic approaches to enhance the survival or functionality of tumor-reactive T cells have the potential to improve the efficacy of cancer immunotherapy. Our previous work identified PTPN22 as a critical regulator of T cell responses to low-affinity antigens (14, 15). PTPN22 inhibits T cell activation by dephosphorylating key TCR-proximal kinases, including Lck and ZAP70, and, consequently, *Ptpn22^–/–^* T cells have a lower threshold for activation (9, 26). In the present work, we showed that *Ptpn22^–/–^* effector OT-1 CTLs had remarkable ability to target and kill ID8 ovarian carcinomas cells expressing the very low-affinity V4 OVA variant both in vitro and in vivo. However, and consistent with previous studies, both control and *Ptpn22^–/–^* effector CTLs were short lived in vivo.

It is generally held that ACT using fully differentiated effector CD8^+^ T cells is inferior to the transfer of memory phenotype T cells for the eradication of tumors (10, 12). It is thought that this phenomenon is related to a reduced capacity of effector T cells to self-renew and survive in vivo. In addition, chronic stimulation of effector T cells by TAAs can lead to functional exhaustion (27). Interestingly, studies showed that PTPN22 deficiency prevented T cell exhaustion in chronic viral infection in mice (28, 29). However, this was predominantly through a cell-extrinsic mechanism as a consequence of reduced type I IFN production by myeloid cells. By limiting PTPN22 deficiency solely to T cells in the current study, cotransfer experiments showed that control and *Ptpn22^–/–^* memory phenotype OT-1 T cells had a similar capacity to form long-lived memory cells upon ACT to tumor-bearing recipients. Most importantly, the enhanced functional capacity of *Ptpn22^–/–^* memory T cells was retained, as evidenced by an improved antitumor response upon transfer to a secondary host. In the current work, we investigated memory cell retention following clearances of tumors expressing high-affinity TAAs. The choice of a high-affinity TAA was to ensure that control and *Ptpn22^–/–^* CD8^+^ T cells were similarly capable of clearing the primary tumor. Using primary tumors expressing a weak TAA complicated analysis of memory cell formation, as we consistently found that ACT of *Ptpn22^–/–^* cells was substantially superior to control ACT in mediating tumor clearance. Nonetheless, we showed that ACT of *Ptpn22^–/–^* but not control memory phenotype T cells 14–24 days prior to tumor inoculation completely prevented growth of low-affinity EL4-OVA-T4 tumors. Protection afforded by *Ptpn22^–/–^* T cells was sustained for at least 3 months, while a population of *Ptpn22^–/–^* T cells could be recovered from hosts for at least 104 days after ACT, indicating the longevity of this population. Therefore, as compared with control cells, long-lived *Ptpn22^–/–^* T cells have superior antitumor activity in response to both high- and low-affinity TAA in vivo.

It is worthy of note that, in our experiments, we did not perform lymphodepletion prior to memory T cell ACT. Host lymphodepletion enhances engraftment of transferred T cells and is associated with favorable ACT therapy outcomes (30, 31) but may cause immune dysfunction (32). Given that we showed previously that, in profoundly lymphopenic environments, *Ptpn22^–/–^* T cells expanded to a greater extent than cotransferred control cells, we did not want to confound the data by lymphodepleting the hosts (14). Despite entering a lymphoreplete host, *Ptpn22^–/–^* memory cells were clearly able to establish themselves and maintain long-term antitumor protection.

In the past decade, numerous studies have determined that T cell activation and differentiation are linked to the regulation of cell metabolism (33). Effector T cells rely on engagement of aerobic glycolysis (34), whereas memory T cells typically use fatty acid oxidation and oxidative phosphorylation (35). We determined that, in our system, memory phenotype OT-1 T cells had enhanced mitochondrial SRC but decreased glucose uptake and glycolytic flux as compared with effector CTLs, consistent with previous literature (21). PTPN22 deficiency did not have a major effect on the engagement of either OXPHOS or glycolytic pathways in either effector or memory phenotype cells. It is worth emphasizing that the differentiation of OT-1 T cells in our experiments was driven by initial stimulation with high-affinity N4 peptide followed by extended culture in high doses of either IL-2 or IL-15. Importantly, PTPN22 does not regulate IL-2 or IL-15 receptor signaling directly. It remains possible that PTPN22 deficiency affects TCR-driven metabolic reprogramming, but this effect is most likely to be apparent upon stimulation with weak agonist peptides (14).

In summary, our work has shown that PTPN22 is a key negative regulator of antitumor T cell responses. The major effect of PTPN22 deficiency is to endow both effector and memory phenotype CD8^+^ T cells with an enhanced capacity to produce inflammatory cytokines and kill tumors expressing low-affinity TAA. Furthermore, as we reported previously, the elevated sensitivity of *Ptpn22^–/–^* cells to TCR triggering may also indirectly affect responses to inhibitory cytokines, such as TGF-β (8). These data provide a strong rationale for targeting PTPN22 expression in T cells for ACT in patients in future studies.

## Methods

### Mice.

OT-1 Ly5.1 *Rag1^–/–^*, *Ptpn22^–/–^* OT-1 Ly5.2 *Rag1^–/–^*, *Ptpn22^–/–^* OT-1 Ly5.2^+/–^Ly5.*1^+/–^Rag1^–/–^*Ubc-GFP, and C57BL/6J mice were maintained under specific pathogen–free conditions at the St. James’s Biomedical Services animal facility at the University of Leeds. Mice were age and sex matched for all experiments. In tumor experiments, recipient mice were randomly assigned to experimental groups. Where applicable, group sizes are indicated by individual data points.

### In vitro T cell differentiation.

For generation of effector CTLs, lymph node OT-1 T cells were stimulated with N4 peptide (Cambridge Peptides) (1 nM) for 2 days in IMDM (Gibco) containing 5% FCS, L-glutamine, antibiotics (penicillin/streptomycin; Gibco), and 50 μM 2-mercaptoethanol. Cells were washed and then expanded and differentiated in the presence of human IL-2 (Peprotech, 20 ng/ml) for a further 4 days. For memory T cell differentiation, anti-CD28 (clone 37.51, BioLegend, 1 μg/mL) was added to culture media during initial N4 stimulation. Cells were differentiated in IL-2 for 2 days and then switched to IL-15 (Peprotech, 20 ng/ml) containing IMDM for the final 2 days of culture.

### Cell lines and tumor models.

ID8 ovarian carcinoma cells expressing OVA variants N4, T4, and V4 were obtained from Dietmar Zehn (Technical University of Munich, Munich, Germany). Firefly luciferase constructs were introduced by lentiviral transduction, as previously described (8). EL4-OVA cells were obtained from Klaus Okkenhaug (University of Cambridge, Cambridge, United Kingdom). Cells were maintained in IMDM containing 5% FCS. EL4-OVA cell cultures were supplemented with 400 μg/ml G418 antibiotic. In some experiments, parental EL4 cells (a gift of Alison Taylor, University of Leeds) were cultured overnight with 1 μM T4 peptide and washed 3 times prior to injection. Adherent ID8 cells were dissociated from culture flasks using trypsin-EDTA (Gibco). Tumor cell suspensions were prepared in PBS (Gibco); EL4-OVA or OVA-T4–loaded parental EL4 cells (1 × 10^6^) were injected s.c. into the right dorsal flank, while ID8 cells (5 × 10^6^) were injected intraperitoneally. 3–5 days after tumor cell injection mice were randomly divided into 3 groups. Control groups received no ACT, while in vitro–generated control or *Ptpn22^–/–^* T cell populations were inoculated i.v., as described in individual figure legends. EL4/EL4-OVA tumor mass was assessed by caliper measurements every 2–3 days, until tumors in control mice reached a maximal diameter of 15 mm. For long-term survival experiments, mice were removed from the experiment when tumors reached 10 mm in diameter. Tumors were resected and wet mass was assessed. In ID8 experiments, tumor bioluminescence was assessed by noninvasive IVIS Spectrum imaging and Living Image Software (Perkin Elmer). For the serial ACT experiments shown in Figure 5, CD8^+^ T cells in host Ubc-GFP LNs and spleens were enriched 14–21 days after ACT by magnetic bead sorting (Miltenyi Biotech). GFP^–^CD8β^+^CD44^hi^ control or *Ptpn22^–/–^* memory T cells were purified to >98% purity using a BD Influx FACS Sorter (BD) and then transferred i.v. to C57BL/6J hosts, prior to tumor cell inoculation.

### In vitro cytotoxicity assay.

Target ID8 cells were seeded in 48 well plates for 4–6 hours, prior to addition of T cells at ratios indicated in figures. Following overnight culture, plates were gently washed in sterile PBS to remove T cells and ID8 cell debris and fresh was IMDM added. Luciferase activity of remaining target cells was assessed by addition of luciferin to wells and IVIS imaging. Specific cell lysis was calculated by comparison of luminescence values in experimental wells to target-only and blank wells.

### Flow cytometry.

The following antibodies were used: CD44-APC Cy7 (clone IM7), CD62L-PE (clone MEL-14), CD8β-PE Cy7 (clone YTS156.7.7), IFN-γ-AF488 (clone XMG1.2), TNF-PerCP Cy5.5 (clone MP6-XT22), Granzyme B-BV421 (clone GB11), Tbet-PE (clone 4B10), CD45.1-AF647 (clone A20), and CD45.2-PE (clone 104) (all from BioLegend). Live/dead aqua dye was from Life Technologies. For analysis of intracellular cytokines, memory phenotype OT-1 T cells or ex vivo LN cells from tumor recipient mice were restimulated with N4 or T4 peptides at concentrations described in figures, in the presence of Brefeldin A (MilliporeSigma) for 4 hours. Cells were labeled with surface and live/dead stains prior to fixation/permeabilization in FoxP3 buffer (eBioscience). In glucose uptake experiments, T cells were cultured in the presence of 50 μM 2-NBDG for 1 hour and then washed 3 times in PBS prior to FACS analysis. Samples were acquired using an LSRII flow cytometer (BD), and data were analyzed using FlowJo software (TreeStar).

### Metabolic analyses.

The Seahorse XFe96 analyser and Mitostress test kits were used to measure metabolic profiles, according to the manufacturer’s instructions. Briefly, effector or memory phenotype T cells (10^5^/well) were transferred to Seahorse assay plates and adhered using a Cell-Tak solution (Corning) in complete XF assay medium. Oligomycin (1 μM), FCCP (1.5 μM), and rotenone/antimycin A (0.5 μM) were injected using the Mitostress test protocol. Data were collected in Wave software and analyzed using GraphPad Prism.

### Western blotting.

T cell lysates were prepared in lysis buffer (0.15 M NaCl, 50 mM Tris-HCL, 1% Triton X-100, 0.5% n-Dodecyl-b-D-maltoside, protease and phosphatase inhibitors), and Western blotting was performed as described previously (36). Membranes were probed with anti-PTPN22 (D6D1H, custom formulation with no carrier protein, Cell Signaling Technology) and ZAP70 (clone 29/ZAP70, BD Biosciences) mAbs. Secondary antibodies were from Molecular Probes. Signals were detected and quantified using a LICOR Imaging System.

### Statistics.

Statistical analyses were performed using GraphPad Prism. Two-tailed Student’s *t* tests, with Holm-Sidak correction for multiple comparisons where appropriate, or 1- or 2-way ANOVA, with Tukey’s multiple comparison test, were applied. For long-term survival experiments, χ^2^ and log-rank tests were applied. *P* < 0.05 was considered significant. Error bars represent SDs. Numbers of experimental and/or technical replicates are described in figure legends.

### Study approval.

Protocols using live animals were performed in accordance with a United Kingdom Home Office project licence (PPL - PDAD2D507) and the regulations of the University of Leeds Animal Welfare and Ethics Review Board, which approved these experiments.

## Author contributions

RJB designed research, performed experiments, acquired and analyzed data, generated figures, and wrote the manuscript. DW performed experiments and analyzed data. RZ provided OT-1hom *Rag1^–/–^* and *Ptpn22^–/–^* OT-1hom *Rag1^–/–^* mice. RJS designed research, performed experiments, acquired and analyzed data, and wrote the manuscript. All authors read and discussed manuscript drafts.

## Supplementary Material

Supplemental data

## Version 1 07/23/2019

In-Press Preview

## Version 2 08/22/2019

Electronic publication
