# Supplementary material for: Deletion of PTPN22 improves effector and memory CD8+ T cell responses to tumors
Source: JCI Insight. 2019 Aug 22;4(16):e127847. doi: 10.1172/jci.insight.127847 (PMC6777814; doi:10.1172/jci.insight.127847)
Supplement: Supplemental data [file jciinsight-4-127847-s089.pdf]

Deletion of PTPN22 improves effector and memory $CD8^+$ T cell responses to tumors

Rebecca J. Brownlie[1,2], David Wright[3], Rose Zamoyska[3], Robert J. Salmond[1,2]

Supplementary Figures 1-3

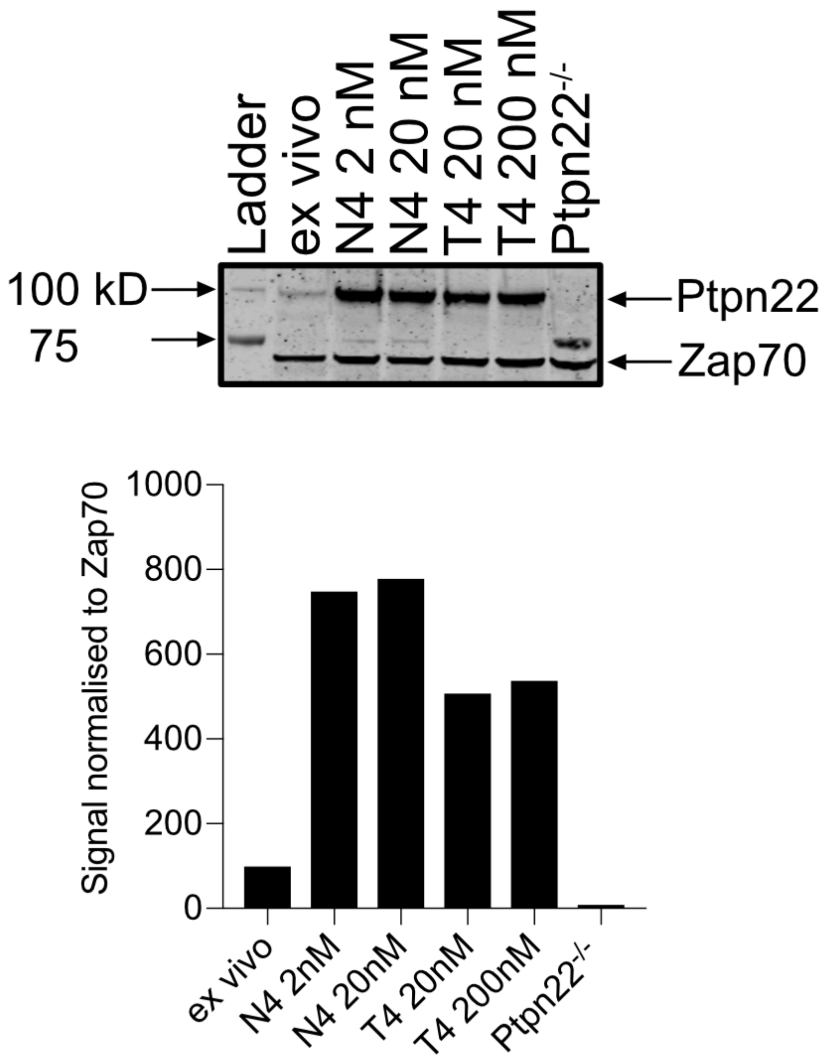

Supplementary Figure 1. Extent of T cell PTPN22 expression following TCR stimulation is dependent upon antigen affinity

Control OT-1 T cells were stimulated with varying concentrations of N4 peptide (high affinity) and T4 (low affinity) for 48hr. Western blots were performed on cell lysates and protein expression of PTPN22 was quantified and normalized to Zap70 expression as shown in the bar chart, using a LICOR imager. *Ptpn22$^{-/-}$* OT-1 T cell lysates served as a negative control. Data are from 1 of 3 repeated experiments.

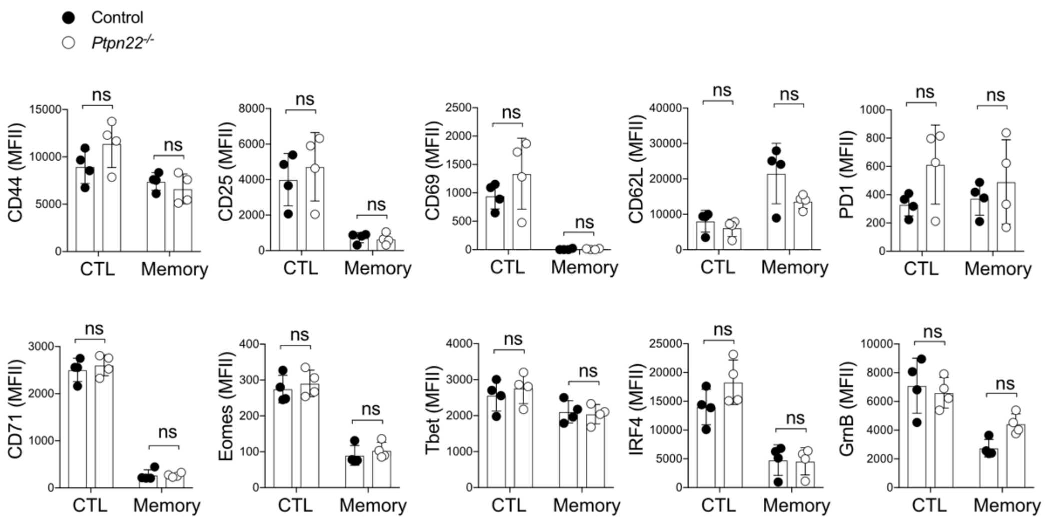

Supplementary Figure 2. FACS analysis of effector and memory phenotype OT-1 T cells

FACS analysis showing mean fluorescence intensity (MFI) of surface and intracellular markers expressed by effector CTL or memory phenotype OT-1 T cells. Dots represent values from individual mice whilst bars represent average values. ns = not significant ($p>0.05$) as determined using Student's *t test* with Holm-Sidak correction for multiple comparisons.

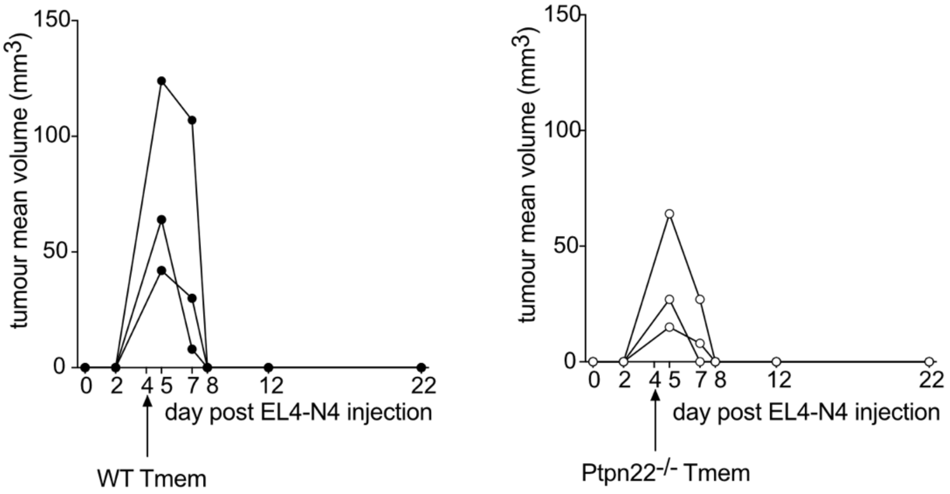

Supplementary Figure 3. WT and *Ptpn22$^{-/-}$* memory phenotype T cells are capable of eradicating established EL4-OVA tumors

Groups of C57BL/6J mice (n=3/group) were injected with $1x10^6$ EL4-N4 cells (d0). On day 4, a time-point at which tumors were palpable, $5x10^6$ *in vitro*-generated control or *Ptpn22$^{-/-}$* T memory phenotype cells were injected i.v. Tumor growth was monitored by caliper measurement throughout the duration of the experiment, as shown in the graphs for individual mice from one experiment (representative of 3 repeated experiments). On day 22 post EL4 injection, mice were culled, and donor OT-1 T cells purified by FACS-sorting for adoptive transfer to naïve hosts, as depicted in Figure 5A.
